# Light‐Triggered In Situ Biosynthesis of Artificial Melanin for Skin Protection

**DOI:** 10.1002/advs.202103503

**Published:** 2022-01-05

**Authors:** Uk‐Jae Lee, Junghyeon Ko, Su‐Hwan Kim, Pyung‐Gang Lee, Young‐Hyeon An, Hyungdon Yun, Dillon T. Flood, Philip E. Dawson, Nathaniel S. Hwang, Byung‐Gee Kim

**Affiliations:** ^1^ School of Chemical and Biological Engineering Institute of Chemical Processes Seoul National University Seoul 08826 South Korea; ^2^ Institute of Molecular Biology and Genetics Seoul National University Seoul 08826 South Korea; ^3^ Department of Chemical Engineering (BK 21 FOUR) Dong‐A University Busan 49315 South Korea; ^4^ Bio‐MAX/N‐Bio Institute of BioEngineerig Seoul National University Seoul 08826 South Korea; ^5^ Department of Systems Biotechnology Konkuk University Seoul 05029 South Korea; ^6^ Department of Chemistry The Scripps Research Institute 10550 N. Torrey Pines Road La Jolla CA 92037 USA; ^7^ Institute for Engineering Research Seoul National University Seoul 08826 South Korea; ^8^ Institute for Sustainable Development(ISD) Seoul National University Seoul 08826 South Korea

**Keywords:** artificial melanosome, *o*‐nitrobenzyl tyrosine, photoactivatable tyrosinase, skin protection, transdermal delivery

## Abstract

Tyrosinase‐mediated melanin synthesis is an essential biological process that can protect skin from UV radiation and radical species. This work reports on in situ biosynthesis of artificial melanin in native skin using photoactivatable tyrosinase (PaTy). The I41Y mutant of *Streptomyces avermitilis* tyrosinase (SaTy) shows enzymatic activity comparable to that of wild‐type SaTy. This Y41 is replaced with photocleavable *o*‐nitrobenzyl tyrosine (ONBY) using the introduction of amber codon and ONBY‐tRNA synthetase/tRNA pairs. The ONBY efficiently blocks the active site and tyrosinase activity is rapidly recovered by the photo‐cleavage of ONBY. The activated PaTy successfully oxidizes L‐tyrosine and tyramine‐conjugated hyaluronic acid (HA_T) to synthesize melanin particles and hydrogel, respectively. To produce artificial melanin in living tissues, PaTy is encapsulated into lipid nanoparticles as an artificial melanosome. Using liposomes containing PaTy (PaTy_Lip), PaTy is transdermally delivered into ex vivo porcine skin and in vivo mouse skin tissues, thus achieving the in situ biosynthesis of artificial melanin for skin tissue protection under UV irradiation. The results of this study demonstrate that this biomimetic system can recapitulate the biosynthetic analogs of naturally occurring melanin. It should therefore be considered to be a promising strategy for producing protective biological molecules within living systems for tissue protection.

## Graphical abstract

Light‐triggered in situ biosynthesis of artificial melanin in native skin is proposed and demonstrated using photoactivatable tyrosinase (PaTy). PaTy is synthesized with site‐specific incorporation of *o*‐nitrobenzyl tyrosine and then encapsulated into lipid nanoparticles as an artificial melanosome for transdermal delivery. This biomimetic system can recapitulate the biosynthetic analogs of naturally occurring melanin.

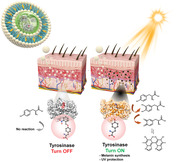

## 1 Introduction

Melanin is a polyphenol‐like biopolymer that shows useful biological activity, such as UV absorption, metal chelation, antioxidant, and radical scavenging properties.^[^ ^1^, ^2^, ^3^, ^4^ ^]^ The primary function of melanin in the human body is to absorb UV radiation, thus protecting the human skin from photo‐aging, mutagenesis, and photo‐carcinogenesis.^[^ ^5^, ^6^ ^]^ Recent research has shown that the transdermal or intracellular delivery of polydopamine nanoparticles can protect cells from UV‐induced DNA damage.^[^ ^7^, ^8^ ^]^ Such melanin‐like nanoparticles are typically synthesized through the autoxidation of dopamine under alkaline conditions,^[^ ^9^ ^]^ thus generating insoluble melanin aggregates that can hamper intracellular delivery into skin cells. Biosynthetic melanin is generally formed in epidermis melanocytes through the tyrosinase‐catalyzed oxidative polymerization of tyrosine, thereby leading to brownish‐black and insoluble eumelanin.^[^ ^10^ ^]^ This melanin's biosynthesis process is an integrated biological system driven by the UV‐induced up‐regulation of tyrosinase gene expression‐which can accelerate melanin biosynthesis‐specifically in melanosomes.^[^ ^11^ ^]^ We speculate that emulating UV‐induced in situ melanin biosynthesis in living skin tissues could be a promising approach to mimicking the biosynthetic process of melanin, unlike the method involving the transdermal delivery of supramolecular melanin particles.

Tyrosinase, a key enzyme in melanin synthesis, is the enzyme that converts L‐tyrosine to L‐3,4‐dihydroxyphenylalanine (L‐DOPA) and its subsequent quinone form, which conjugates with neighboring amine, thiol, or phenolic groups to form melanin. Our group has previously established a *Streptomyces avermitilis‐*derived tyrosinase (SaTy)‐mediated hydrogel formation system for tissue engineering.^[^ ^12^, ^13^, ^14^, ^15^ ^]^ SaTy can easily oxidize tyrosine residues at the surface of proteins or polysaccharides because it has a flat surface, wide substrate entrance, and a short distance from the surface to the active site compared to tyrosinases from other organisms.^[^ ^14^, ^16^, ^17^ ^]^ We have demonstrated that this SaTy‐based hydrogel system shows excellent potential as a tissue adhesive and material for tissue regeneration.^[^ ^13^, ^14^ ^]^ Researchers have also attempted to achieve the spatiotemporal control of dynamic biological processes through the modulation of enzymatic activity with external stimuli such as UV irradiation.^[^ ^18^ ^]^ In particular, photoactivatable enzymes can theoretically be designed using amber codon suppression and the incorporation of photocleavable unnatural amino acid, such as *o*‐nitrobenzyl tyrosine (ONBY), in the active site by suppressor tRNA.^[^ ^18^, ^19^, ^20^, ^21^, ^22^, ^23^ ^]^ However, the fact that the active site of tyrosinase contains a di‐copper scaffold makes it difficult to select proper mutation sites for the insertion of ONBY, since a minute misalignment of copper ions can result in an irreversible loss of activity.^[^ ^16^ ^]^ Therefore, to our knowledge, no studies have reported photoactivatable tyrosinase (PaTy).

Herein, we designed and synthesized PaTy that enabled UV‐induced in situ melanin biosynthesis for skin protection in living tissues. To synthesize PaTy in *Escherichia coli* (*E. coli*), we utilized: i) SaTy containing amber codon; and ii) suppressor tRNA / ONBY‐tRNA synthetase (ONBYRS) pair from *Methanocaldococcus jannaschii*. PaTy has ONBY instead of a tyrosine residue at the I41 position, where ONBY can physically interrupt the binding of the substrate (i.e., L‐tyrosine) into the active site. As UV irradiation caused *o*‐nitrobenzaldehyde to be rapidly removed from ONBY, the tyrosinase activity was mostly recovered. We confirmed the potential usage of PaTy for several biomedical applications, such as spatiotemporally controlled hydrogel formation and UV protection through in situ melanin biosynthesis in living tissue. Through ex vivo and in vivo tests, we successfully confirmed its transdermal delivery and UV protection by mimicking melanosomes with liposomes containing PaTy. This study demonstrated a promising model system wherein the in situ on‐demand modulation of enzyme activity through UV irradiation enables the cross‐linking of biomacromolecules or melanin synthesis in a living system for tissue engineering.

## 2 Results and Discussion

**Figure 1:**
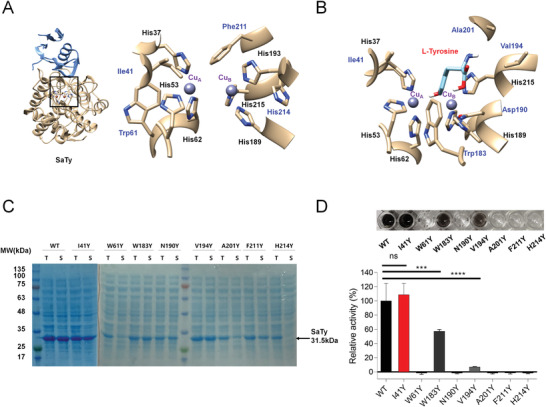
Target residues for photoactivation were selected through a structural analysis and mutation study of SaTy. A) Amino acid residues within 4.65 Å from copper ions. B) Amino acid residues within 4 Å from substrate L‐tyrosine. The black‐colored letters indicate copper‐holding histidines. C) SDS‐PAGE analysis showing mutant tyrosinase expression in *E. coli* BL21(DE3). “T” and “S” respectively indicate total fraction (insoluble + soluble) and supernatant fraction (soluble) after cell lysis. D) Relative activity of mutant tyrosinases compared against wild‐type (WT) activity (100%). Reaction volume 200 µL includes 50 × 10^–3^ m Tris‐HCl buffer (pH 8.0), 1 × 10^–3^ m L‐tyrosine, 10 × 10^–6^ m CuSO_4_, and the same amount of mutant tyrosinase soluble fraction. Data presented as mean and standard deviation (mean ± SD), *n* = 3, *p*‐values are calculated using one‐way ANOVA. ns, non‐significant; ***p < 0.001; ****p < 0.0001.

We first analyzed the crystal structure of SaTy (PDB: 6J2U) to select possible insertion sites for ONBY instead of tyrosine, while considering the length of the *o*‐nitrobenzyl group (4.71‐6.02 Å) as a critical parameter for predicting the validity of the replacement. We selected eight candidates in total to be replaced with ONBY, including four amino acid residues (Ile41, Trp61, Phe211, and His214) located within a distance of 4.65 Å from the two type III copper ions, and five amino acid residues (Ile41, Trp183, Asp190, Val194, and Ala201) located within a distance of 4 Å from whole atoms of the docked L‐tyrosine substrate (**Figure** **1**A,B); note that Ile41 is included in both groups. These eight sites in SaTy were individually mutated with tyrosine, a photocleaved form of ONBY (Table S1, Supporting Information). Then, their expression levels, protein solubilities, and relative enzyme activities were examined (Figure 1C,D). Only mutant SaTy I41Y showed an expression level, a solubility, and an enzymatic activity that were comparable to those of the SaTy wild‐type. Other SaTy mutants showed significantly decreased activities (W183Y and V194Y) or loss of function (W61Y, N190Y, A201Y, F211Y, and H214Y) (Figure 1D and Figure S1, Supporting information). The protein structures of SaTy I41Y and I41ONBY were respectively generated using the software Chimera and Avogadro, and ligand binding was compared using AutoDock Vina. The results of the comparison between the SaTy I41Y and I41ONBY protein models suggested that the additional *o*‐nitrobenzyl moiety of ONBY located above the copper ion (Cu_A_) could interfere with L‐tyrosine substrate binding (Figure S2, Supporting Information). The protein model showed that the insertion of ONBY at the SaTy I41 position blocked the active site, thus causing the substrate tunnel of SaTy I41ONBY (7.7 Å) to be five times that of SaTy I41Y (1.43 Å) (Table S2, Supporting Information). Hence, I41 was selected as the site for ONBY incorporation.

**Figure 2:**
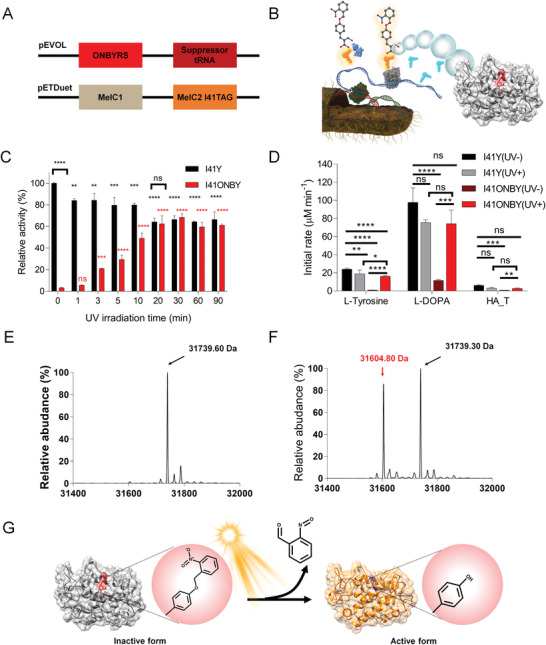
Synthesis and photoactivation tests of PaTy were evaluated with and without UV irradiation. A) Two vectors were constructed for PaTy synthesis. B) Schematic illustration describing PaTy synthesis with unnatural amino acid incorporation. C) Relative enzymatic activities of I41Y and I41ONBY mutants were measured according to UV irradiation time. Data presented as mean ± SD, *n* = 3, *p*‐values are calculated using one‐way ANOVA. ns, non‐significant; **p < 0.01; ***p < 0.001; ****p < 0.0001. D) Initial rates of I41Y and I41ONBY were assessed according to UV irradiation for 30 min with various substrates (L‐tyrosine, L‐DOPA, and HA_T). Data presented as mean ± SD, *n* = 3, *p*‐values are calculated using one‐way ANOVA. ns, non‐significant; *p < 0.05; **p < 0.01; ***p < 0.001; ****p < 0.0001. E) Average molecular mass of purified PaTy and F) UV‐irradiated PaTy for 30 min, which revealed the cleavage of ONBY from PaTy. G) Schematic illustration of PaTy photoactivation describing the release of ONBY from PaTy under UV irradiation.

Using a recombinant *E. coli* BL21(DE3) system, it was confirmed that ONBY‐incorporated SaTy was successfully expressed at the level of 5.3 mg L^−1^ through optimization of the IPTG concentration, medium selection, and *E. coli* codon usage (**Figure** **2**A,B and Figures S3 and S4, Supporting Information). The expressed SaTy I41ONBY was almost inactive, but its tyrosinase activity was recovered upon UV irradiation, thus confirming that SaTy I41ONBY was converted into SaTy I41Y. The initial rate of SaTy I41ONBY showed only 3% of the SaTy I41Y mutant, having activity comparable to that of SaTy wild‐type. Melanin synthesis was not observed until several hours of the reaction. As the duration time of UV irradiation was extended, SaTy I41ONBY accordingly showed increased tyrosinase activity, resulting in SaTy I41Y. The recovery of the tyrosinase activity was equivalent to 68.4% of SaTy I41Y, and the increase in activity was maximized after 30 min of UV irradiation (Figure 2C and Figure S5, Supporting Information). The fully recovered tyrosinase activity of SaTy I41ONBY was 21‐fold higher than that of the initial tyrosinase activity of SaTy I41ONBY. However, the enzyme activity of SaTy I41ONBY gradually decreased when UV irradiated for over 30 min.

Next, the SaTy I41Y and I41ONBY mutants were compared in terms of substrate specificity, kinetic parameters, and mass change upon optimal UV irradiation (Figure 2D–G). Photoactivation of SaTy I41ONBY, so called PaTy, was observed with a monophenol compound (i.e., tyrosine), a diphenol compound (i.e., DOPA) and a tyramine modified hyaluronic acid (i.e., HA_T) (Figure 2D). Specifically, its activity for monophenolic compounds (L‐tyrosine and HA_T) was tightly controlled by the photoactivation of PaTy. By contrast, the catechol oxidase activity of PaTy for L‐DOPA was still observed, which might indicate that the incorporation of ONBY did not completely interfere with type III copper coordination for enzyme catalysis. PaTy had a higher *K*_m_ value than SaTy I41Y, indicating that the *o*‐nitrobenzyl moiety somewhat hindered the substrate binding of the enzyme, as was predicted (**Table** **1**). Further, the incorporation and cleavage of ONBY in PaTy were successfully confirmed by high‐resolution mass spectrometry. Upon 30 min of UV irradiation, 46.3% of ONBY41 in PaTy was converted into Y41 (Figure 2E–G and **Table** **2**). In addition, since incorporating unnatural amino acids such as ONBY could lead to an increase in thermal stability, the thermal stability of PaTy was estimated.^[^ ^24^ ^]^ *T*_m_ of PaTy (61.06 °C) was increased by over 5 °C compared to that of SaTy (Table S3, Supporting Information). The enhanced thermal stability protected PaTy from denaturation by the heat generated during UV irradiation.

**Table 1:** Kinetic parameters of tyrosinase mutants I41Y and I41ONBY evaluated for substrates L‐tyrosine and L‐DOPA. Values of *k*_cat_ and *K*_m_ were obtained with non‐linear regression by Sigma plot 10.0 software. Data presented as mean ± SD, *n* = 5

	L‐Tyrosine			L‐DOPA			Mono/di
	*k*_cat_ [s^−1^]	*K*_m_ [ 10^–6^ m]	*k*_cat_ /*K*_m_ [m ^−1^s^−1^]	*k*_cat_ [s^−1^]	*K*_m_ [10^–6^ m]	*k*_cat_ /*K*_m_ [m ^−1^s^−1^]	
I41Y (UV−)	0.85 ± 0.003	70.7 ± 0.00	1.2 × 10^4^	4.90 ± 0.94	564 ± 230	8.7 × 10^3^	1.38
I41Y (UV+)	0.67 ± 0.04	139.7 ± 36.74	4.8 × 10^3^	3.57 ± 0.38	418 ± 108	8.5 × 10^3^	0.56
I41ONBY (UV−)	N.D^a)^	N.D	N.D	0.84 ± 0.45	1513 ± 1213	5.6 × 10^2^	N.D
I41ONBY (UV+)	0.56 ± 0.05	199.8 ± 61.14	2.8 × 10^3^	4.96 ± 1.42	1080 ± 512	4.6 × 10^3^	0.61

^a)^ not determined.

**Table 2:** Average molecular weights of mutant tyrosinases

	Observed mass^a)^ [Da]	Expected mass^b)^ [Da]	ΔMass^c)^ [Da]
SaTy I41Y	31 604.00	31 605.06	1.06
SaTy I41ONBY (UV−)	31 739.60	31 740.06	0.46
SaTy I41ONBY (UV+)	31 739.30	31 740.06	0.76
	31 604.80	31 605.06	0.26

^a)^ Observed mass was generated by LC‐ESI‐TOF. Deconvolution was conducted by MaxEnt1;

^b)^ Expected mass was calculated based on the amino acid sequence of tyrosinases using the Expasy computational web server;

^c)^ ΔMass was the absolute number obtained by subtracting the observed mass from the expected mass.

**Figure 3:**
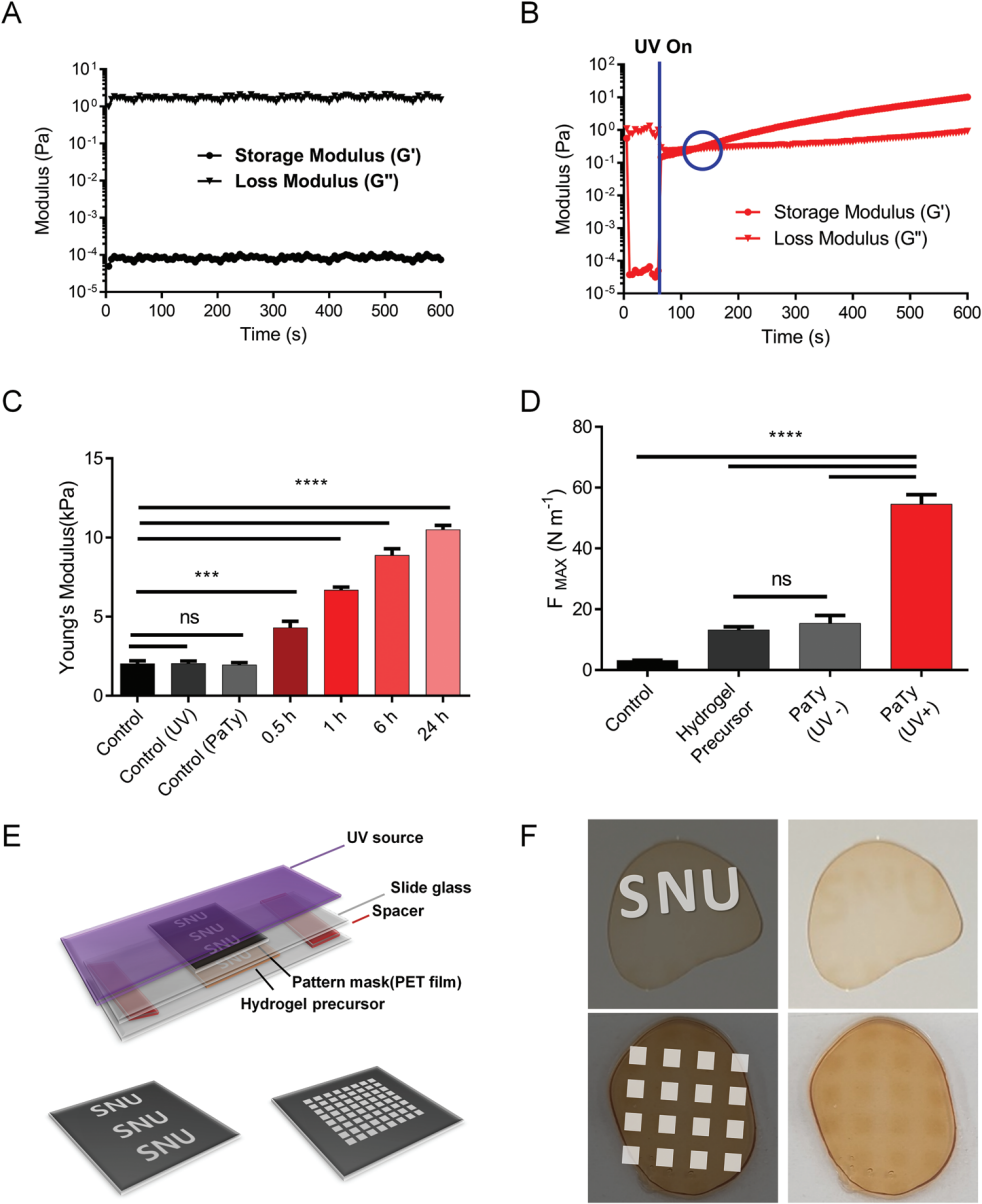
In situ hydrogel formation was induced by UV irradiation onto PaTy. Rheological analysis to determine the gelation time A) without UV irradiation or B) with UV irradiation. The crossover point of G′ (storage modulus) and G″ (loss modulus) indicates the gelation point. C) Young's modulus of hydrogel precursor with PaTy was measured according to UV irradiation time. Data presented as mean ± SD, *n* = 4, *p*‐values are calculated using one‐way ANOVA. ns, non‐significant; ***p < 0.001; ****p < 0.0001. D) The hydrogel with UV‐activated PaTy had the maximum adhesive force (*F*_MAX_) compared to other groups. Data presented as mean ± SD, *n* = 3, *p*‐values are calculated using one‐way ANOVA. ns, non‐significant; ****p < 0.0001. E) Schematic illustration showing that patterned hydrogel can be fabricated using both PaTy and photomask, and F) letters “SNU” and dotted pattern were observed after UV irradiation.

From the previous studies, we have reported that SaTy can cross‐link hyaluronic acid conjugated with phenolic moieties for applications in tissue engineering.^[^ ^17^, ^25^ ^]^ To demonstrate that PaTy derived from SaTy can also induce HA_T crosslinking, we evaluated the hydrogel formation of the phenolic hydrogel by UV‐induced PaTy activation (**Figure** **3**). Three different properties of UV‐induced enzymatic cross‐linked hydrogels were evaluated upon UV irradiation: i) viscoelastic properties (Figure 3A,B); ii) cross‐link density (Figure 3C); and iii) tissue adhesiveness (Figure 3D). Under physiological conditions, inactive PaTy could not form hydrogel (Figure 3A). However, upon UV irradiation, G′ (storage modulus) and G″ (loss modulus) were crossed over within 1 min (Figure 3B), indicating that PaTy could be activated in a very short time. Photo‐crosslinkable hydrogel has been applied to hydrogel patterning (Figure 3E,F) and hydrogel‐mediated embolization (Figure S6, Supporting Information). Using a simple photo‐mask, we could easily generate hydrogel patterns ranging from the dot pattern to complex letters. Although we could only demonstrate the embolization in a microfluidic system, we expect that if the endoscope is incorporated with a UV light source, this system can serve as an alternative to conventional materials for aneurysm embolization such as coils or liquid materials.^[^ ^26^ ^]^ This demonstrates that UV inducible PaTy can be applied in additional biomedical applications.

**Figure 4:**
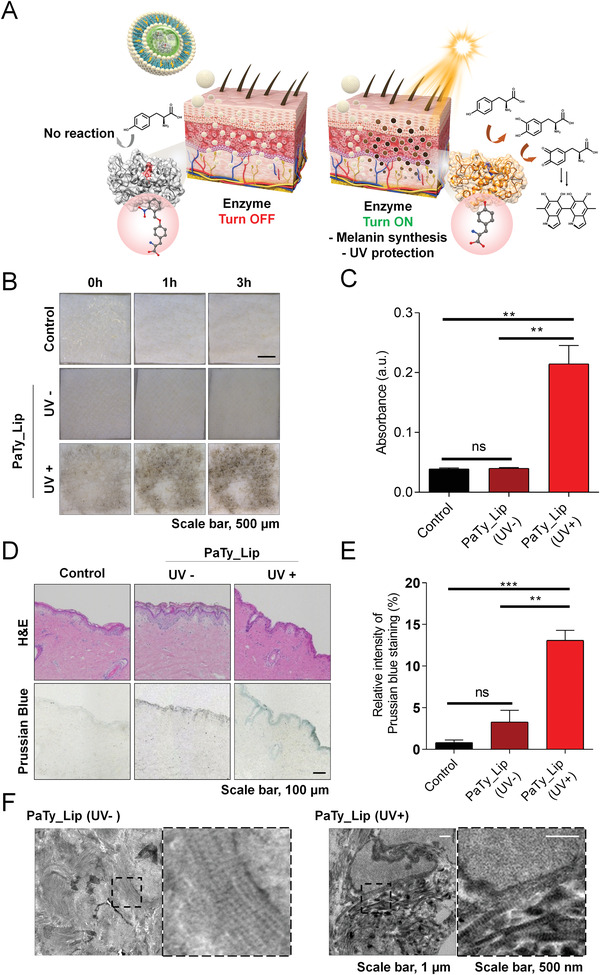
Ex vivo PaTy_Lip penetration and melanin synthesis were evaluated using porcine skin. A) Schematic illustration shows in situ synthesis of artificial melanin from UV‐mediated activation of PaTy. B) Photographs show ex vivo skin penetration and melanin synthesis by period (0, 1, and 3 h after delivery and UV irradiation). C) Synthesized melanin was extracted from the skin tissue and quantified by measuring the absorbance at 475 nm. Data presented as mean ± SD, *n* = 3, *p*‐values are calculated using one‐way ANOVA. ns, non‐significant; **p < 0.001. D) The skin tissue was stained with Hematoxylin and eosin (H&E) staining and Prussian blue staining at 3 h post‐treatment, and E) Relative intensity of melanin in Prussian blue staining images was evaluated. Data presented as mean ± SD, *n* = 3, *p*‐values are calculated using one‐way ANOVA. ns, non‐significant; **p < 0.001; ***p < 0.0001. F) TEM images reveal that melanin was strongly formed with UV irradiation in porcine skin.

Without any enhancers or carriers, the transdermal delivery of protein or other drugs larger than 500 Da is hampered by the lamellar structure of the lipid and keratin in human skin.^[^ ^27^ ^]^ To deliver PaTy (MW: 31.7 kDa) into living skin tissues, we designed artificial melanosomes with liposomes encapsulating PaTy (called PaTy_Lip). We hypothesized that the delivered PaTy_Lip under UV‐irradiated conditions could activate melanin formation using endogenous L‐tyrosine in the skin (**Figure** **4**A). The fabricated PaTy_Lip showed an average of diameter of 134.8 ± 10.6 nm with 59% encapsulation efficiency (Figure S7 and Table S4, Supporting Information). PaTy was observed in the PaTy_Lip group, but no protein was shown in empty liposome group (Empty_Lip) in SDS‐PAGE analysis (Figure S7B, Supporting Information). PaTy_Lip showed a larger particle size compared to that of Empty_Lip (Figure S7C,D, Supporting Information). We confirmed the biocompatibility of PaTy_Lip with mouse fibroblast (NIH3T3) and human dermal fibroblast (HDF). For both cells, there were no significant decreases in cell viability between PaTy_Lip concentrations of 10 and 25 µg mL^−1^, while the NIH 3T3 cell metabolism began to decrease when the concentration of PaTy_Lip was over 100 µg mL^−1^ (Figure S8, Supporting Information). Using an ex vivo porcine skin, we assessed the delivery efficacy of PaTy_Lip as well as its melanin‐forming ability (Figure 4B–F). UV irradiation (16.6 mW cm^−2^, 30 min) on porcine skin with PaTy_Lip treatment induced an immediate change in color to black, while no color change was observed in the control group or the group with PaTy_Lips treatment without UV irradiation (Figure 4B). In quantitative studies, PaTy_Lip with UV irradiation showed more than a fivefold increase in melanin synthesis compared to that without UV irradiation (Figure 4C). These results suggest that PaTy_Lip was delivered effectively through both the epidermal and dermal layers. Only the skin with UV irradiated PaTy_Lip showed dense Prussian blue staining in histology (Figure 4D,E) as well as a black area in the transmission electron microscopy (TEM), thus image indicating melanin synthesis (Figure 4F). In our ex vivo analysis, we utilized an excessive amount of L‐tyrosine and PaTy for clear visualization and hyperpigmentation of the skin. Even though hyperpigmentation is evident as a result of PaTy dependent activation and formation of artificial melanin, hyperpigmentation could be controlled with the optimal delivery amount of PaTy_Lip. Furthermore, we hypothesize that natural exfoliation of skin and proliferation of skin epithelial cells would eventually remove the artificial melanin formed within the skin.

**Figure 5:**
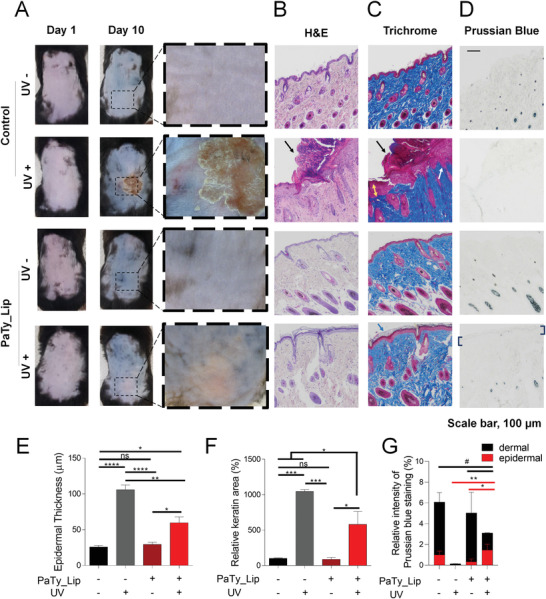
In vivo UV protection effect of artificial melanin was evaluated. A) Photographs show that the skin was damaged from UV exposure, whereas the PaTy_Lip‐treated skin was not. B) H&E staining reveal that there was little alteration of PaTy_Lip‐treated skin. By contrast, epidermal thickening and scab formation occurred in the control group. C) Trichrome staining reveal that UV‐treated skin suffered from orthokeratosis (blue arrow), acanthosis (yellow arrow), and rete pegs (white arrow). D) Prussian blue staining display melanin formation in the epidermal regions (bracket) by PaTy_Lip as well as hair follicle melanin formation in dermal regions with less UV damaged skin. E) Epidermal thickness; Data presented as mean ± SD, *n* = 7, *p*‐values are calculated using one‐way ANOVA. ns, non‐significant; *p < 0.05; **p < 0.001; ****p < 0.0001; and F) Relative area of keratin indicate that the PaTy_Lip‐treated skin showed less UV‐induced damage. Data presented as mean ± SD, *n* = 3, *p*‐values are calculated using one‐way ANOVA. ns, non‐significant; *p < 0.05; ***p < 0.001. G) Relative intensities of melanin in epidermal and dermal regions of Prussian blue staining images were measured, including epidermal melanin by PaTy_Lip and dermal melanin by hair follicle. Data presented as mean ± SD, *n* = 4, *p*‐values are calculated using one‐way ANOVA. *, compared with epidermal PaTy_Lip (UV+); #, compared with dermal PaTy_Lip (UV+); ns, non‐significant; *#*p* < 0.05; **p < 0.01.

To verify the UV protection effect of PaTy_Lip in living tissues, PaTy_Lip (0.17 mg cm^−2^) was applied to in vivo skin (C57BL/6 mice) with UV irradiation (50 mW cm^−2^, 1 h). The applied dose of PaTy_Lip was determined by the cytotoxicity level of the particle when it penetrated through dermis and plasma (Figure S8, Supporting Information).^[^ ^28^, ^29^ ^]^ There were no significant differences in epithelial thickness between the control group and the PaTy_Lip‐treated group without UV irradiation. By contrast, under UV irradiation, the skin in the control group showed epidermal hypertrophy with scabs and other signs of UV damage, such as acanthosis and keratosis with prominent rete pegs, along with increased epithelial thickness (≈116 µm) in histological analysis (**Figure** **5**A–D).^[^ ^30^, ^31^ ^]^ However, the PaTy_Lip‐treated mice showed dramatically reduced signs of UV damage in the skin. For one, the skin had a reduced epithelial thickness (≈67 µm). It also showed significant decreases in the percentages of keratin, orthokeratosis, acanthosis, and rete pegs (Figure 5E,F). More specifically, the increase in keratin content caused by UV irradiation induces keratosis pilaris with skin irritation.^[^ ^30^ ^]^ For this reason, less keratosis was observed in Masson's Trichrome staining of the PaTy_Lip group, indicating that PaTy_Lip successfully protected skin tissues from UV damage.

We also performed Prussian blue staining to confirm whether the UV protection effect could be attributed to PaTy‐induced melanin formation (Figure 5D,G). We could not observe any melanin formation in the epidermis region of PaTy_Lip‐treated mice without UV irradiation. However, there was a significant increase in melanin formation in the epidermis region of PaTy_Lip‐treated mice under UV irradiation. The skin under PaTy_Lip protection from UV showed reduced disturbance of follicular melanogenesis. On the other hand, melanogenesis was fully impaired without PaTy_Lip.^[^ ^32^ ^]^ The melanin observed in human skin has a sun protection factor (SPF) of 1.5–2.0, thus providing 33–50% UV protection.^[^ ^3^ ^]^ However, melanin synthesized by PaTy_Lip had 2.9 sun protection factor (SPF) with 65.5% sun protection. In addition, the melanin synthesized by PaTy_Lip covered a UV protection range including both UVB and UVA (**Table** **3** and Figure S9, Supporting Information).

**Table 3:** UV protection effect of melanin synthesized by PaTy and PaTy_Lip was evaluated using the conventional method,^[^ ^33^ ^]^ revealing higher SPF and *λ*_critical_ in melanin formed by PaTy_Lip than those in the control group, with PaTy_Lip showing a similar UV protection effect to human skin melanin

	Control	PaTy	PaTy_Lip
SPF^a)^	0.38	0.94	2.91
*λ*_critical_ [nm]^b)^	362	384	386
UVA/UVB	0.19	0.68	0.90

^a)^ SPF: sun protection factor;

^b)^ *λ*_critical_: critical wavelength.

UV irradiation on skin can form pyrimidine dimers or cyclobutane pyrimidine dimers (CPD) in the damaged DNA sites and induce phosphorylation of H2AX to form *γ*H2AX at DNA double‐strand breaks.^[^ ^34^, ^35^, ^36^ ^]^ The immunohistochemical evaluation showed that cyclobutane pyrimidine dimers (CPD) and *γ*H2AX were much higher in control mice than in PaTy_Lip‐treated mice with UV‐irradiation (Figure S10, Supporting Information). These results confirmed that melanin synthesis induced by transdermally delivered PaTy_Lip successfully reduced UV damage in vivo. Overall, our system successfully protected the skin from UV damage in vivo from the DNA to tissue levels, thus indicating its potential use as an in situ UV protector.

## 3 Conclusion

In summary, we proposed and demonstrated artificial melanosome with a biomimetic system of melanin biosynthesis in a living system. PaTy was constructed by incorporating photolabile ONBY into the Y41 site in SaTy I41Y mutant. We efficiently inactivated SaTy by hindering its substrate‐binding site without directly interfering with di‐copper coordination. With simple UV irradiation, PaTy could successfully recover the tyrosinase activity without any deformation or significant activity loss. Further, PaTy_Lip was successfully delivered into mice skin with lipid nanoparticles. The PaTy delivered in this way was able to efficiently synthesize melanin particles in mouse skin in vivo. In vivo analysis using C57BL/6 mice revealed that PaTy_Lip in epidermis induced melanin formation, thus providing effective protection from UV damage to the skin tissues. Consequently, newly designed PaTy could broaden the horizon of recapitulating biological processes for tissue engineering.

## Conflict of Interest

The authors declare no conflict of interest.

## Supporting information

Supporting Information

## Data Availability

The data that support the findings of this study are available from the corresponding author upon reasonable request.
