# Supplementary material for: Light‐Triggered In Situ Biosynthesis of Artificial Melanin for Skin Protection
Source: Adv Sci (Weinh). 2022 Jan 5;9(7):2103503. doi: 10.1002/advs.202103503 (PMC8895148; doi:10.1002/advs.202103503)
Supplement: Supplementary file 1 — Supporting Information [file ADVS-9-2103503-s001.pdf]

WILEY-VCH

ADVANCED SCIENCE
Open Access

Supporting Information

for *Adv. Sci.*, DOI: 10.1002/advs.202103503

Light-Triggered *In Situ* Biosynthesis of Artificial Melanin for Skin Protection

Uk-Jae Lee, Junghyeon Ko, Su-Hwan Kim, Pyung-Gang Lee, Young-Hyeon An, Hyungdon Yun, Dillon T. Flood, Philip E. Dawson, Nathaniel S. Hwang, and Byung-Gee Kim**

Supporting Information

Light-Triggered *In Situ* Biosynthesis of Artificial Melanin for Skin Protection

Uk-Jae Lee[#], Junghyeon Ko[#], Su-Hwan Kim[#], Pyung-Gang Lee, Young-Hyeon An, Hyungdon Yun, Dillon T. Flood, Philip E. Dawson, Nathaniel S. Hwang, and Byung-Gee Kim**

Experimental Section

Materials

L-tyrosine, L-3,4-dihydroxyphenylalanine, asolectin from soybean, Triton X-100, and cholesterol were purchased from Sigma-Aldrich (Yongin, Korea). Restriction enzymes, T4-DNA ligase, DNA polymerase, reagents for genetic engineering, phosphate-buffered saline (PBS), and snakeskin dialysis tubing were purchased from Thermo Fisher Scientific (Seoul, Korea). Chloroform was purchased from Dae Jung (Siheung, Korea). Tyrosinase gene of *Streptomyces avermitilis* MA4680 (ATCC 31267) was sequenced as published previously.[1, 2] Ni-NTA agarose (nickel-charged resin for His-tag purification) was purchased from Qiagen Korea Ltd. (Seoul, Korea). Tyrosinase body protein gene, *MelC2,* was newly synthesized by LnC Bio (Seoul, Korea). Primers were synthesized by Bionics (Seoul, Korea).

Mutation site screening for photoactivatable tyrosinase

For the structure of tyrosinase, PDB 6J2U in Protein Data Bank was used.[1] The structure of the L-tyrosine substrate was generated by Chemdraw and the the energy was minimized by Chem 3D. L-tyrosine docking substrate was generated using AutoDock Vina software according to the manufacturer's instructions.[3] The grid size was x = 30, y = 30, and z = 30. The grid center was located at x = 30.008, y = 72.35, and z = 4.707. The constructed structure was visualized using Chimera software.[4] The structure of the I41Y mutant was generated using the rotamer option in Chimera with a Dunbrack force field and a possibility of 62.2%. The I41ONBY mutant was constructed in Avogadro software and the energy was minimized with a general AMBER force field (GAFF).[4, 5] The distances from copper ions and substrate were both calculated using Chimera software.

Site-directed mutagenesis of tyrosinase

Escherichia coli strain BL21(DE3) was transformed with plasmid pETDuet encoding *MelC1* and *MelC2* from *Streptomyces avermitilis* with a T7 promoter and an ampicillin resistance marker. The construction of the pETDuet vector containing *MelC1* and *MelC2* was described in a previous work.[1] In this study, the codon-optimized *MelC2* gene of tyrosinase was synthesized by LnCBio (Seoul, Korea) and inserted into the pETDuet vector containing *MelC1* using NcoI and KpnI restriction enzymes. The plasmid was purified with Exprep™ (GeneAll). Herculase II fusion DNA polymerase (Agilent, California, USA) was used. The PCR conditions for primers were a primary denaturation at 96 °C for 10 min, 25 cycles of denaturation at 96 °C for 1 min, annealing at 45, 55, or 65 °C for 40 sec, and extension at 72 °C for 3 min 30 sec; the final extension was held at 72 °C for 5 min. Upon completion of the PCR reaction, purification was conducted with Expin™ (GeneAll). FastDigest DpnI (Thermo Scientific, Seoul, Korea) was used to remove the template plasmid.

Construction of photoactivatable tyrosinase

The previously constructed pETDuet::*MelC1*::*MelC2* was purified from *E. coli* BL21(DE3). In the pETDuet vector, mutagenesis PCR of *MelC2* was conducted to provide an amber codon (TAG) at the I41 site as described above. The pEVOL vector with six suppressor tRNAs was generously donated by Hyun-Soo Lee from Sogang University.[6] *Methanocaldococcus jannaschii* tyroyl-tRNA synthetase for *o*-nitrobenzyl tyrosine (ONBYRS) was obtained by conducting several site-directed mutagenesis PCR reactions from DOPA synthetase gene with the primers listed in **Table S1**. ONBYRS was inserted into the pEVOL vector using BglII/SalI and NdeI/PstI restriction sites to construct pEVOL::*ONBYRS*::*suptRNA*. The construct was transformed into *E. coli* BL21(DE3) through heat shock at 42 °C with two plasmids for 1 min 30 sec. The transformant was then inoculated onto a Luria-Bertani (LB) plate containing ampicillin (100 μg mL^{-1}) and chloramphenicol (20 μg mL^{-1}).

Synthesis and purification of photoactivatable tyrosinase

Transformed cells were amplified in 4 ml of LB media at 37 °C with ampicillin (100 μg mL^{-1}) and chloramphenicol (20 μg mL^{-1}). These cells were inoculated into 200 ml of LB media containing ampicillin (100 μg mL^{-1}) and chloramphenicol (20 μg mL^{-1}). When the OD_{600} approached 0.8, $CuSO_4$ (1 mM), *o*-nitrobenzyl tyrosine (1 mM), isopropylthio-β-galactoside (IPTG) (0.05 mM), and arabinose (1.33 mM) were added to the media. Cells were grown to saturation at 18 °C, after which they were harvested by centrifugation at 4000 rpm at 4 °C for 10 min and washed with 50 mM Tris-HCl buffer (pH 8.0). These cells were then lysed with a sonicator (VCX750 Sonic Vibra Cell). Total fraction and soluble fraction were collected and subjected to sodium dodecyl sulfate-polyacrylamide gel electrophoresis (SDS-PAGE) analysis with 12% acrylamide gel. The protein marker was MG 3-color protein marker. General His-tag purification was applied as described in previous studies.[1, 7, 8] Buffer exchange was conducted using an Amicon Ultra 10K centrifugal filter with 50 mM Tris-HCl buffer (pH 8.0), then centrifuged at 4,000 rpm for 10 minutes several times.

Assay of photoactivatable tyrosinase activity

The concentration of the purified protein was measured by the Bradford assay. Next, 200 nM of tyrosinases in 50 mM of Tris-HCl (pH 8.0) was UV irradiated using an XX-15L A UV bench lamp at 16.6 mW cm^{-2} (5.3 mW) for the indicated time periods (1, 3, 5, 10, 20, 30, 60, and 90 min) at 4 °C. The intensity of the UV irradiation was measured using the NOVA II from OPHIR (California, USA). Tyrosinase activity was measured using 200 nM tyrosinase, 10 μM $CuSO_4$, and 1 mM L-tyrosine in 50 mM of Tris-HCl buffer (pH 8.0). The initial rate of enzymatic activity was determined by measuring the concentration of dopachrome ($\varepsilon_{475\ dopachrome}$ = 3600) at 475 nm or 3-methyl-2-benzothiazolineone

hydrazone hydrochloride (MBTH) adduct product ($\varepsilon_{505\ MBTH\ adduct}$ = 39000) at 505 nm using a Spec Star Nano plate reader at 37 °C.

Mass analysis of intact protein by high-resolution mass spectrometry

To produce tyrosinase for mass analysis, 1 mM of $ZnSO_4$ was added during the synthesis of protein instead of $CuSO_4$ in an attempt to prevent cross-linking between proteins. Purified proteins were diluted with mass spectrometry-grade water (0.1 mg mL^{-1}). A 5 μL (ca. 500 ng) sample was analyzed on a Waters I-Class liquid chromatograph (LC) connected to a Waters G2-XS time of flight (TOF) mass spectrometer. Liquid chromatography was conducted using a Waters BEH C18 column (2.1 x 55 mm, 1.7 μm, 300 Å) with an isocratic gradient of 0.4 mL min^{-1} held for 0.4 minutes, which was followed by a 5 – 99% B gradient over 4.6 minutes (A: 0.1% formic acid in water; B: 0.1% formic acid in acetonitrile). The mass spectroscopy and data processing conditions were ESI + ionization (m/z 500 - 2000). Spectra were combined across the main peak area, with deconvolution to a neutral mass achieved using a Waters MaxEnt1. The removal of N-terminal methionine was considered in the calculation of the average molecular mass.

Fabrication of hydrogel with photoactivatable tyrosinase (PaTy)

Tyramine-conjugated hyaluronic acid (HA_T) and EGCG dimer-conjugated hyaluronic acid (HA_E) were synthesized using previously reported methods.[7, 8] Hydrogel precursors were prepared by dissolving either HA_T (4% w/v) or HA_E (2% w/v) in distilled water. Both solutions were sterilized through syringe-driven filtration (Acrodisc® Syringe Filter 0.2 μm Supor® Membrane, PALL Life Science, New York, USA). For rheologic analysis upon UV irradiation, rheological tests were conducted in Demo lab (Anton Paar Korea) using a rheometer (MCR 302, measuring cell: P-

PTD & H-PTD 200; measuring System: PP 25, Anton-Paar, Austria). Samples were loaded onto the plate and data were measured in terms of the storage modulus G' and loss modulus G" at 37 °C according to UV irradiation (300 mW). The crossover point of G' and G" indicated critical strain. To fabricate hydrogel with a pattern, PET films were printed in an SNU letter pattern and a dot pattern. The hydrogel precursor was located between the glass using a spacer; it was covered with the printed PET film. A UV lamp was placed 5 cm above the hydrogel precursor and irradiated at 300 mW intensity.

Fabrication of photoactivatable tyrosinase-encapsulated liposomes (PaTy_Lip)

Liposomal encapsulated PaTy (PaTy_Lip) was fabricated using the conventional lipid film hydration method. Cholesterol and phospholipid were dissolved at a 2 : 8 ratio in chloroform. The chloroform was then removed with a rotary evaporator at 45 °C for 10 min. The remaining solvent was completely removed using a vacuum pump overnight. The lipid film was formed and hydrated in 5 mL of phosphate-buffered saline (PBS) solution with 10 μM PaTy. PaTy solution and lipid film were vortexed for 15 min to form liposome particles. To produce particles with even sizes, ultrasonication (Sonics & Materials, Inc., Newtown, USA) was conducted for 15 min with 30% amplitude and a pulse speed of 5 sec pulse/5 sec rest. Residuals were removed using a 3.5 kDa dialysis membrane for two days. Empty liposome (Empty_Lip) was synthesized with previously described method without addition of PaTy and dialysis process. The particle size and zeta potential were measured using a dynamic light-scattering device (Zetasizer Nano ZS, Malvern, United Kingdom). To analyze with SDS-PAGE, PaTy_Lip was synthesized with 30 μM of PaTy and loaded in 12% acrylamide SDS-PAGE gel with PaTy and Empty_Lip. To calculate the encapsulated efficiency, Triton X-100 was added to disassemble the lipid layer. The amount of enzyme was calculated and compared with the amount of PaTy solution added to lipid film using BCA assay. The

encapsulation efficiency (EE%) was then determined by inputting the relevant data into the following equation:

$$EE\% = \frac{enzyme\ encapsulated}{enzyme\ added} \times 100$$

After extrusion through a 0.22 μm syringe filter, the particles were collected.

***Ex-vivo* transdermal particle delivery test**

To assess skin penetration, micropig skin (2 cm x 2 cm x 400 μm, APRUES Co.) was prepared in humidified conditions at 37 °C. The test was conducted with a control group without UV (UV -), a PaTy_Lip group without UV (UV -), and a PaTy_Lip group with UV (UV +). To evaluate the penetration depth and enzymatic activity with naked eyes, skin samples were soaked in L-tyrosine solution (20 mM) for 30 min. As an enhancer, a microneedle roller (1.5 mm, KYRIBE) was applied 10 times. To begin, particle solution of 200 μL was applied to the micropig skin. After 10 min, the remaining solution on the skin was removed. Next, the skin was UV irradiated with 16.6 mW cm^{-2} for 30 min. Melanin in the skin was then collected using a tissue protein extraction reagent (T-PER) and quantified by measuring the absorbance at 475 nm with a UV-spectroscope (TECAN Infinite M200 Pro, Männedorf, Switzerland). Histology was evaluated by H&E and Prussian blue staining (ferrous ion uptake reaction). H&E staining was performed according to conventional methods. To detect melanin in the skin, Prussian blue staining of melanin was performed. Paraffin-sectioned skins were de-paraffinized through three changes of xylene for 3 min each. After re-hydration, the sections were placed in 2.5% ferrous sulfate solution for 1 h. These sections were washed wtih six changes of distilled water for 3 min each. They were then placed in 1% potassium ferricyanide in 1% acetic acid for 30 min. After being washed with four changes of water for 3 min each, the samples were dehydrated and a mounting solution was applied. The intensity of Prussian blue staining was quantified using ImageJ software. TEM images were obtained by cutting the skin into a 1-mm-

diameter cylindrical shape. Samples were primarily fixed with Karnovsky's fixative at 4 °C overnight. Post-fixation proceeded at 4 °C with 1% osmium tetroxide. En bloc staining was performed overnight at 4 °C with 0.5% uranyl acetate. The samples were first treated with ethanol and Spurr's resin mixture. Next, they were put into Spurr's resin overnight at 4 °C and then moved to room temperature for 3 h. Finally, the sample preparation was completed by transferring the samples to a 70 °C oven for polymerization. These pretreated samples were cut using an ultra-thin micro-cutter (Ultramicrotome EM UC7, Wetzlar, Leica). TEM images were taken using a Biotransmission Electron Microscope (120 kV, Energy-Filtering Transmission Electron Microscope LIBRA 120, Carl Zeiss, Oberkochen, Germany).

UV protection ability of PaTy and PaTy_Lip

To measure the sun protection factor (SPF), critical wavelength (λ_c), and UVA/UVB ratio, 50 μL of either PaTy or PaTy_Lip was mixed with 2 mM tyrosine solution and each material was diluted to 200 μg mL^{-1} in ethanol. The absorbance was then measured. SPF was calculated using the following formula:

$$SPF_{spectrophotometic} = CF \times \sum_{290}^{320} EE(\lambda) I(\lambda) Abs(\lambda)$$

Where CF is the correction factor (=10), EE (λ) is the erythemal effect spectrum, I (λ) is the solar intensity spectrum, and Abs (λ) is the absorbance of the solution. The values of EE and I were determined using the reference method.[9] The Abs (λ) value was measured by UV-spectroscopy. The critical wavelength value and UVA/UVB ratio were calculated using the following equations:

$$\int_{290}^{\lambda_c} A(\lambda)\, d\lambda \ = 0.9 \int_{290}^{400} A(\lambda) d\lambda$$

and

$$\frac{UVA}{UVB} ratio = \frac{\int_{320}^{400} A(\lambda)\, d\lambda \Big/ \int_{320}^{400} d\lambda}{\int_{290}^{320} A(\lambda)\, d\lambda \Big/ \int_{290}^{320} d\lambda}$$

Where A (λ) was the average absorbance at each wavelength.

Biocompatibility test of PaTy_Lip

Prior to PaTy_Lip treatment, mouse fibroblast (NIH3T3) and human dermal fibroblast (HDF) were seeded into 24-well plates at a density of $1x10^4$ cells/well a day. PaTy_Lip at different concentrations (10, 25, 50, and 100 μg mL^{-1}) was added to each well. Live/Dead assay was performed after one day. PrestoBlue assay was conducted at different time intervals (up to 3 days) at 37 °C with 5% CO_2. The effect of PaTy_Lip treatment on cell viability was measured using a LIVE/DEAD® cell viability kit (Thermo Fisher Scientific). The effect of PaTy Lip treatment on cell proliferation was measured using the PrestoBlue assay (Thermo Fisher Scientific). As controls, cells without particles and cell medium alone were assayed. After different incubation times, cells were washed in PBS and once in a mixture of Dulbecco's Modified Eagle Medium (DMEM) and PrestoBlue cell viability reagent (900 μL DMEM + 100 μL PrestoBlue). Next, the mixture of DMEM and PrestoBlue cell viability reagent was added to different wells and incubated at 37 °C in the dark for 3 h. Finally, the fluorescence intensity of the reduced resazurin product was assessed at an excitation wavelength at 570 nm and an emission wavelength of 600 nm, using a UV-spectroscope (TECAN Infinite M200 Pro). Normalized cell viability was calculated as follows:

$$Normalized\ cell\ viability\ (\%) = \frac{experimental\ value - cell\ medium\ control\ value}{cells\ without\ particle\ value - cell\ medium\ control\ value} \times 100$$

In vivo skin penetration and UV protection analysis

All animal tests and experimental procedures were approved by the Institutional Animal Care and Use Committee at Seoul National University (#SNU-201229-2). The subjects of this study consisted of male C57BL/6 mice at 8 weeks old. On the day before the experiment, the animals were shaved. As the *ex vivo* experiment, a microneedle roller was applied as an enhancer 10 times. The particle solution (200 μL, 0.17 mg cm^{-2}) was applied every day. The solution that did not enter was wiped off after 10 min. UV was applied for 1 h with an intensity of 50 mW cm^{-2} at 395 nm using a hand-held UV lamp (Ultrafire WF-501B). The same process was repeated for 10 days. Mice were euthanized after the application on the last day. Histology was evaluated by H&E and Prussian blue staining as described in the *ex vivo* experiment. Masson's trichrome staining (MTC staining) was performed with a conventional method. Epidermal thickness was calculated by dividing the epidermal area by the contour length. The epidermal thickness, keratin area, and intensity of Prussian blue stain were quantified using ImageJ software.

Immunohistochemical (IHC) staining

The damage induced by UV and the protection effect of PaTy_Lip were compared through IHC staining of cyclobutane pyrimidine dimers (CPD) and γH2AX with mice used in the *in vivo* experiment. Paraffin sectioned skin was de-paraffinized and hydrated. Proteinase K with Tris-EDTA buffer (pH 8.0) was treated for 20 min in a humidified condition at 37 °C. After PBS washing, 10% goat serum (NGS) was used for blocking for 1 h at room temperature. The tissue samples were then stained overnight at 4 °C with primary antibody (CPD, Kamiya#MC-062; γH2AX, Abcam#ab26350) diluted in goat serum. The samples were washed with PBS and then incubated with Alexa 488 goat anti-mouse antibody IgG (Invitrogen) with NGS. Next, the samples were mounted with DAPI

(Abcam#ab104139) and examined with an inverted microscope (NIKON ECLIPSE Ti2-E). The intensities of DAPI, CPD, and γH2AX staining were measured using ImageJ software.

Statistical Analysis

Data were processed as described in Experimental Section. Values (over $n = 3$) were shown as mean and standard deviation (mean ± SD). Data were analyzed with the Student's t-test or one-way ANOVA with Prism 8 software. Data indicated with P-value as ns (not significant), * ($p < 0.05$), ** ($p < 0.01$), *** ($p < 0.001$), **** ($p < 0.0001$).

Supplementary Figures

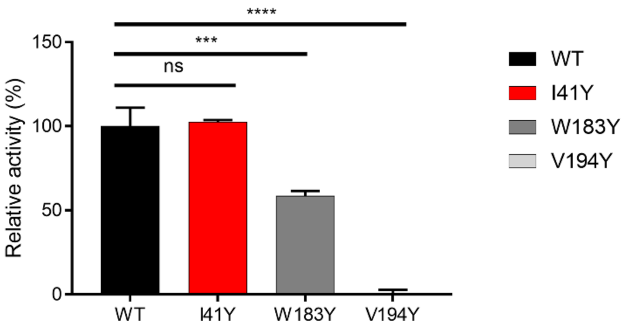

Figure S1. Relative enzymatic activities of purified wild-type (WT), mutant I41Y, W183Y, and V194Y tyrosinase. Enzyme activity of WT tyrosinase was expressed as 100% of relative activity. Reaction volume of 200 μL contained 50 mM Tris-HCl buffer (pH 8.0), 1 mM L-tyrosine, 10 μM $CuSO_4$, and 200 nM of purified mutant tyrosinase. Data presented as mean ± SD, $n = 3$, P-values are calculated using one-way ANOVA, ns, non-significant; ***$p < 0.001$; ****$p < 0.0001$.

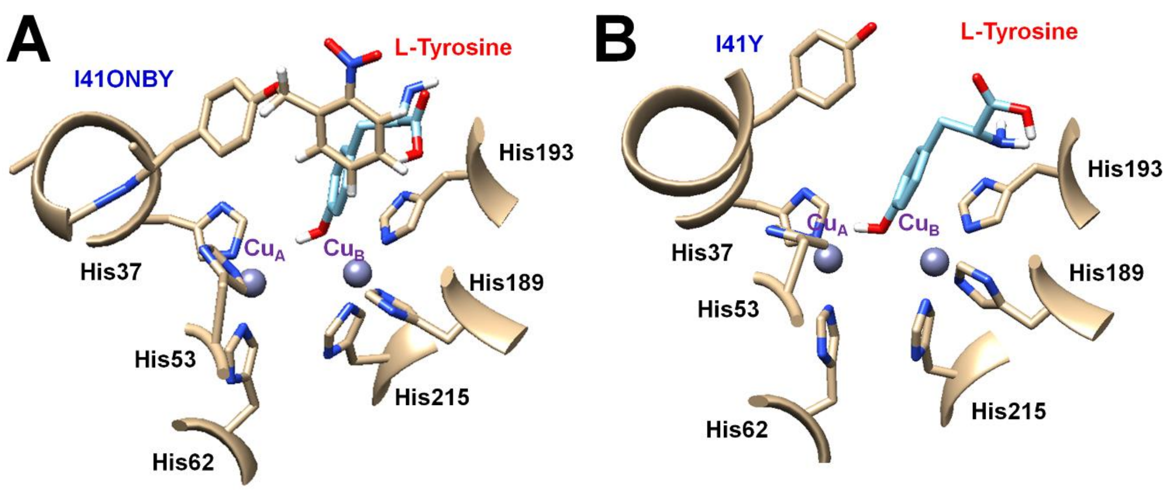

Figure S2. Computationally generated protein structures around active sites of I41ONBY and I41Y. (A) I41ONBY model generated with Avogadro software; its energy was minimized with a GAFF force field. (B) I41Y model generated by Chimera software with a 62.2% possibility.

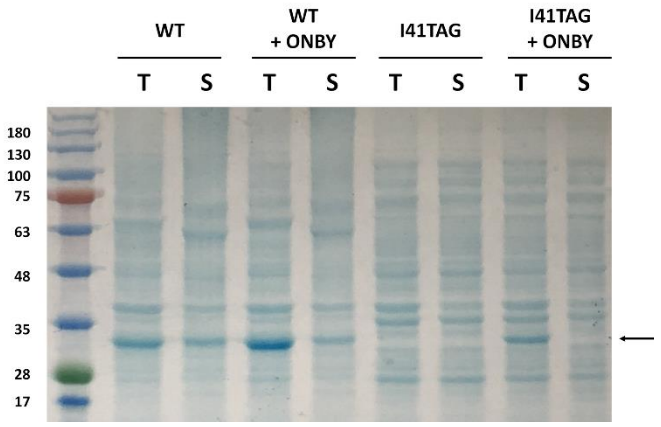

Figure S3. SDS-PAGE analysis of the wild-type and I41TAG mutant tyrosinase evaluated according to the addition of *o*-nitrobenzyl tyrosine (ONBY) expressed in *E. coli* BL21(DE3). "T" and "S" respectively indicate total fraction (insoluble + soluble) and a supernatant fraction (soluble) after cell lysis. The arrow at the right of the SDS-PAGE gel indicates the size of tyrosinase.

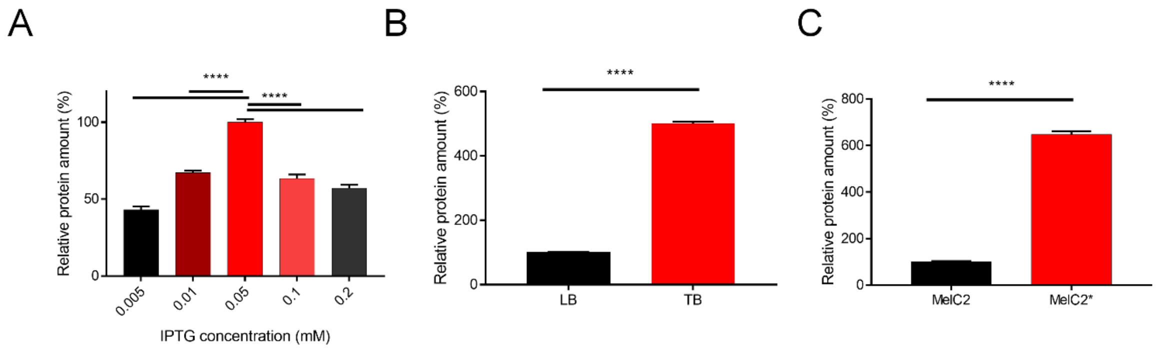

Figure S4. Tyrosinase production optimized with IPTG concentration, media selection, and codon optimization. **(**A) Relative protein amounts induced with various IPTG concentrations (0.005, 0.01, 0.05, 0.1, and 0.2 mM). Data presented as mean ± SD, $n = 3$, P-values are calculated using one-way ANOVA. ****$p < 0.0001$. (B) Relative protein amounts compared in the same amounts of culture media of Luria-Bertani (LB) broth and Terrific broth (TB). Data presented as mean ± SD, $n = 3$, P-values are calculated using Student's t-test. ****$p < 0.0001$. (C) Relative protein amounts compared upon codon optimization. MelC2* indicates the codon-optimized gene inserted instead of the wild-type MelC2. Data presented as mean ± SD, $n = 3$, P-values are calculated using Student's t-test, ****$p < 0.0001$.

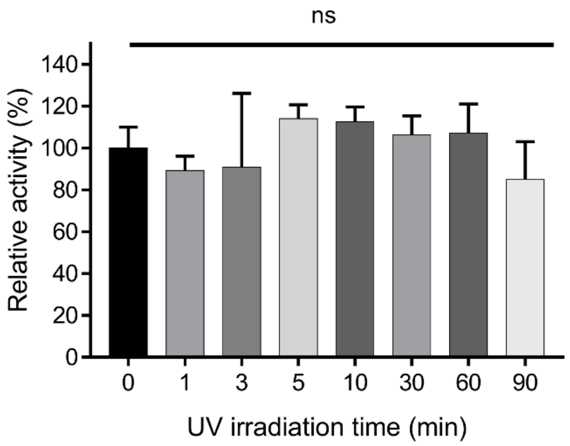

Figure S5. Relative activities of wild-type SaTy at different UV irradiation time points. Reaction volume of 200 μL contained 50 mM Tris-HCl buffer (pH 8.0), 1 mM L-tyrosine, 10 μM $CuSO_4$, and 200 nM of mutant tyrosinase. Data presented as mean ± SD, $n = 3$, P-values are calculated using one-way ANOVA. ns, non-significant.

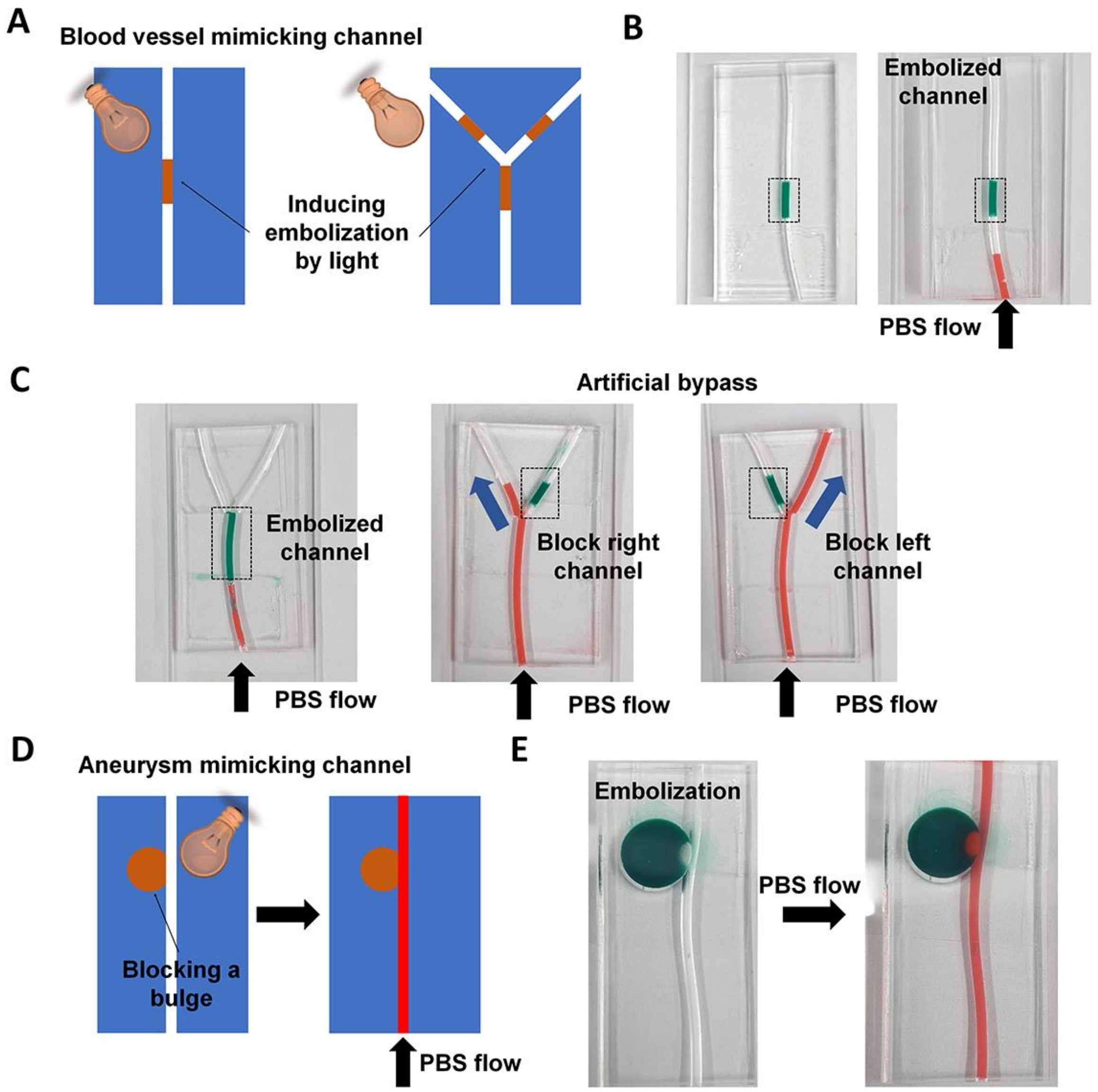

Figure S6. PaTy-induced spatiotemporal hydrogel formation that shows potential for embolization. (A) Schematic illustration describing the embolization channel experiment with the channel. (B) A single channel was blocked with embolized hydrogel upon UV irradiation. (C) Y-shaped channel was blocked with embolized hydrogel upon UV irradiation at the center of the channel (left), the right channel (center), and the left channel (right). (D) Schematic illustration of the embolization experiment with bulge. (E) The bulge was filled with embolized hydrogel. PBS, phosphate-buffered saline.

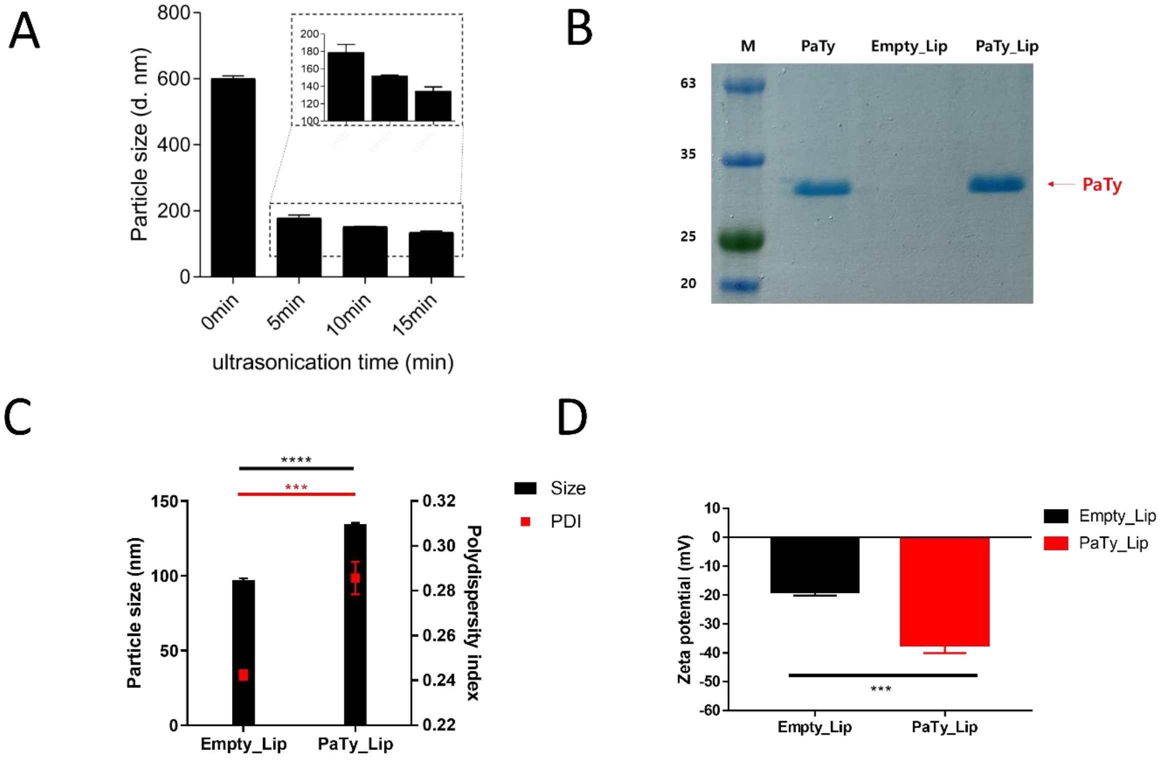

Figure S7. Characterization of PaTy_Lip proceeded with SDS-PAGE analysis and dynamic light scattering (DLS) instrument. (A) Particle sizes of PaTy_Lip were measured according to ultrasonication time. Particle size variation was accomplished by changing ultrasonication time (0, 5, 10, and 15 min) under the same operation conditions (pulse rate 5 sec pulse/5 sec rest, 30% amplitude). Data presented as mean ± SD, n = 3. (B) According to SDS-PAGE analysis, PaTy was observed only in the PaTy_Lip samples and no protein was observed in the Empty_Lip samples. (C) The PaTy_Lip displayed a bigger particle size than those of Empty_Lip. Data presented as mean ± SD, n = 3, P-values are calculated using Student's t-test, ***$p < 0.001$; ****$p < 0.0001$. (D) The surface charge of PaTy_Lip was compared with those of Empty_Lip. Data were presented as means ± SD, n = 3, P-values are calculated using Student's t-test, ***$p < 0.001$.

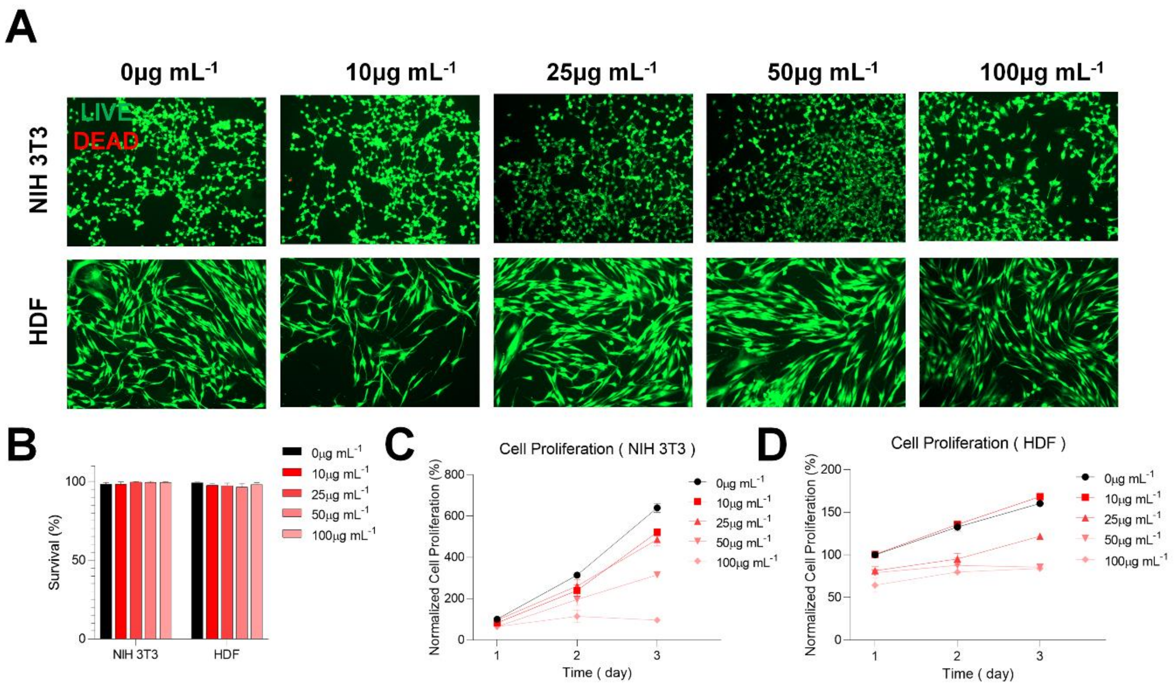

Figure S8. PaTy_Lip biocompatibility was evaluated with NIH 3T3 and HDF. (A) Live and Dead images at 24 h after cell seeding show no cytotoxicity with PaTy_Lip in different concentrations (0, 10, 25, 50, and 100 μg mL^{-1}) (B) Images of Live and Dead staindend cells were quantified. Data presented as mean ± SD, *n* = 4. (C, D) Cell metabolism changes over 3 days with different concentrations of PaTy_Lip (0, 10, 25, 50, and 100 μg mL^{-1}) were evaluated with PrestoBlue assay. Data presented as mean ± SD, *n* = 3.

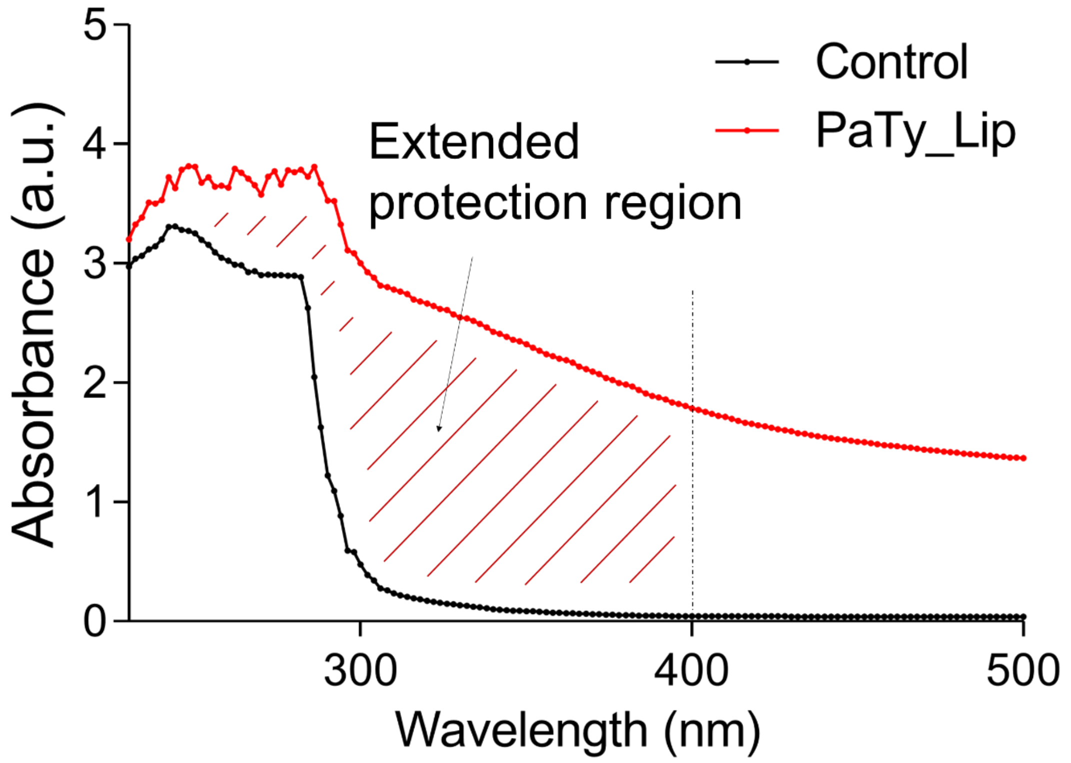

Figure S9. UV protection effect of melanin synthesized by PaTy_Lip was evaluated by absorbance measurement in the UV region (280-400 nm). Control is ethanol solution without PaTy_Lip. Wavelength ranges of 280-315 nm and 315-400 nm respectively indicate UVB and UVA.

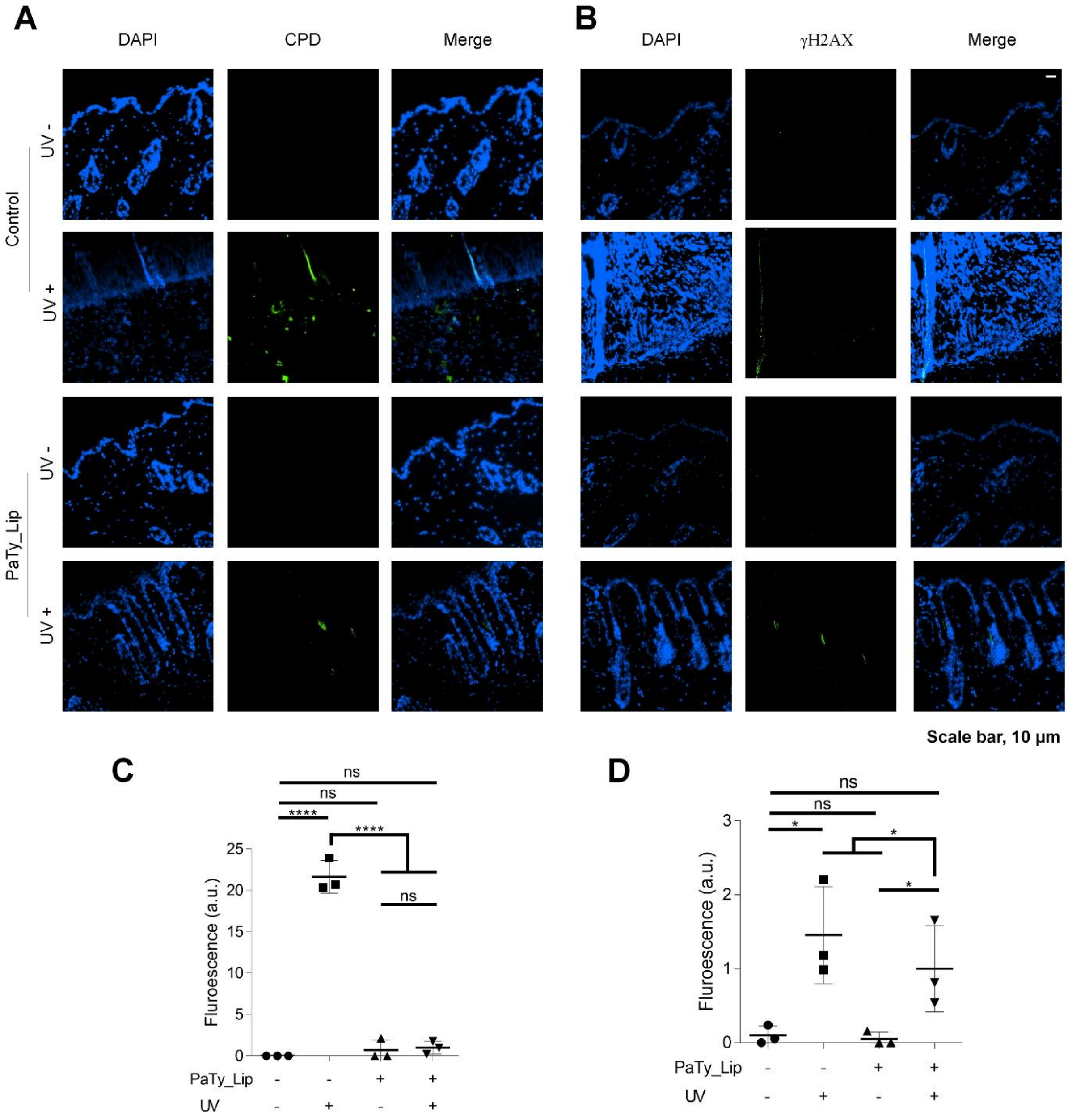

Figure S10. UV protection was evaluated based on delivered PaTy_Lip *in vivo* with immunohistochemistry. (A) CPD staining and (B) γH2AX staining of mouse skin after UV irradiation with or without PaTy_Lip were compared. Relative fluorescence of (C) CPD and (D) γH2AX on skin were quantified showing UV protection effect of PaTy_Lip. Data presented as mean ± SD, $n = 3$. ns, non-significant; *$p < 0.05$; **$p < 0.01$; ***$p < 0.001$; ****$p < 0.0001$.

Supplementary Tables

Table S1. List of primers used for photoactivatable tyrosinase synthesis.

Primer Name	Sequence
W61Y_F[a)]	CCG AGC TTT CTT CCG TAT CAT CGT CGC TTC CTG
W61Y_R	CAG GAA GCG ACG ATG ATA CGG AAG AAA GCT CGG
W183Y_F	AAT CAC CTG GAA GGG TAT CGT GGA GTT AAT TTA
W183Y_R	TAA ATT AAC TCC ACG ATA CCC TTC CAG GTG ATT
N190Y_F	GGA GTT AAT TTA CAT TAT CGT GTG CAT GTT TGG
N190Y_R	CCA AAC ATG CAC ACG ATA ATG TAA ATT AAC TCC
V194Y_F	CAT AAT CGT GTG CAT TAT TGG GTT GGT GGG CAA
V194Y_R	TTG CCC ACC AAC CCA ATA ATG CAC ACG ATT ATG
A201Y_F	GTT GGT GGG CAA ATG TAT ACT GGC GTG AGC CCG
A201Y_R	CGG GCT CAC GCC AGT ATA CAT TTG CCC ACC AAC
F211Y_F	CCG AAT GAT CCC GTG TAT TGG TTA CAT CAT GCT
F211Y_R	AGC ATG ATG TAA CCA ATA CAC GGG ATC ATT CGG
H214Y_F	CCC GTG TTT TGG TTA TAT CAT GCT TAT ATT GAT
H214Y_R	ATC AAT ATA AGC ATG ATA TAA CCA AAA CAC GGG
I41Y_F	ACA CAT AAT GCT TTT TAT ATG GGT GAT ACG GAT
I41Y_R	ATC CGT ATC ACC CAT ATA AAA AGC ATT ATG TGT
I41TAG_F[b)]	ACA CAT AAT GCT TTT TAG ATG GGT GAT ACG GAT
I41TAG_R	ATC CGT ATC ACC CAT CTA AAA AGC ATT ATG TGT

D_25Lys_F[c)]	TTA AGA GAG GTT TTA AAA AAA GAT GAA AAA TCT
D_25Lys_R	AGA TTT TTC ATC TTT TTT TAA AAC CTC TCT TAA
65G67A70H_F	GGA TTT GAT ATA ATT ATA GGC TTG GCT GAT TTA CAC GCC TAT TTA AAC CAG AAA GG
65G67A70H_R	CCT TTC TGG TTT AAA TAG GCG TGT AAA TCA GCC AAG CCT ATA ATT ATA TCA AAT CC
F108E_F	GTT TAT GGA AGT GAA GAA CAG CTT GAT AAG GAT
F108E_R	ATC CTT ATC AAG CTG TTC TTC ACT TCC ATA AAC
158S162E167Q_F	CAA TAA TGC AGG TTA GCG ATA TTC ATT ATG AAG GCG TTG ATG TTG CAG TTG GAG GGA TGG AGC A
158S162E167Q_R	TGC TCC ATC CCT CCA ACT GCA ACA TCA ACG CCT TCA TAA TGA ATA TCG CTA ACC TGC ATT ATT G

[a)]Tyrosinase mutation to tyrosine; [b)]Tyrosinase mutation to amber codon; [c)]tRNA synthetase mutation to change *o*-nitrobenzyl tyrosine.

Table S2. Tunnel analysis of mutant tyrosinases. Tunnel of tyrosinase was calculated based on the optimal distance from the copper ions to the surface using CAVER 3.0.

	Bottleneck radius (Å)	Tunnel length (Å)	Tunnel curvature
SaTy WT	2.07	1.96	1.002
SaTy I41Y	2.07	1.43	1.114
SaTy I41ONBY	1.92	7.70	1.153

Table S3. Thermal stability of mutant tyrosinases. Data presented as mean ± SD. n = 5.

	SaTy WT	SaTy I41Y	SaTy I41ONBY
T_m (°C)	57.97 ± 0.44	55.58 ± 0.94	61.06 ± 0.85

Table S4. Optimization of PaTy_Lip particle size. Data presented as mean ± SD. n = 3.

Enzyme concentration (μM)	1	2	5	10
Particle size (nm)	132.4 ± 1.4	124.1 ± 0.9	131.5 ± 1.6	134.8 ± 0.6
Zeta potential (mV)	-33.9 ± 2.8	-36.3±3.3	-35.3 ± 3.4	-34.2 ± 2.7
Encapsulation efficiency (%)	69.1	74.6	56.0	59.0
Polydispersity index (PDI)	0.42 ± 0.02	0.26 ± 0.01	0.34 ± 0.01	0.29 ± 0.01

References

[1] N. Lee, S.-H. Lee, K. Baek, B.-G. Kim, *Applied microbiology and biotechnology* **2015**, *99*, 7915.

[2] N. Lee, E. J. Kim, B.-G. Kim, *ACS chemical biology* **2012**, *7*, 1687.

[3] O. Trott, A. J. Olson, *Journal of computational chemistry* **2010**, *31*, 455.

[4] E. F. Pettersen, T. D. Goddard, C. C. Huang, G. S. Couch, D. M. Greenblatt, E. C. Meng, T. E. Ferrin, *Journal of computational chemistry* **2004**, *25*, 1605.

[5] M. D. Hanwell, D. E. Curtis, D. C. Lonie, T. Vandermeersch, E. Zurek, G. R. Hutchison, *Journal of cheminformatics* **2012**, *4*, 17.

[6] T. S. Young, I. Ahmad, J. A. Yin, P. G. Schultz, *Journal of molecular biology* **2010**, *395*, 361.

[7] S.-H. Kim, K. Kim, B. S. Kim, Y.-H. An, U.-J. Lee, S.-H. Lee, S. L. Kim, B.-G. Kim, N. S. Hwang, *Biomaterials* **2020**, 119905.

[8] S.-H. Kim, S.-H. Lee, J.-E. Lee, S. J. Park, K. Kim, I. S. Kim, Y.-S. Lee, N. S. Hwang, B.-G. Kim, *Biomaterials* **2018**, *178*, 401.

[9] R. M. Sayre, P. P. Agin, G. J. LeVee, E. Marlowe, *Photochemistry and Photobiology* **1979**, *29*, 559.
